# NEDD4 Induces K48-Linked Degradative Ubiquitination of Hepatitis B Virus X Protein and Inhibits HBV-Associated HCC Progression

**DOI:** 10.3389/fonc.2021.625169

**Published:** 2021-03-09

**Authors:** Tao Wan, Zhao Lei, Biao Tu, Tianyin Wang, Jiale Wang, Feizhou Huang

**Affiliations:** Department of Hepatobiliary Surgery, The Third Xiangya Hospital of Central South University, Changsha, China

**Keywords:** NEDD4, hepatocellular carcinoma, HBx, HBV-associated HCC, ubiquitin-proteasome pathway

## Abstract

Neural precursor cell expressed developmentally downregulated gene 4 (NEDD4) plays two opposite roles in carcinogenesis. It has been reported that NEDD4 inhibits hepatocellular carcinoma (HCC) progression; however, little is known about its potential function and molecular mechanism in HCC in the context of hepatitis B virus (HBV) infection. In this study, we analyzed NEDD4 expression in 199 HCC specimens with or without HBV infection and observed that NEDD4 expression was unrelated to HBV exposure in HCC tumor tissue but that high NEDD4 expression conferred better overall survival (OS) and progression-free survival (PFS) than low NEDD4 expression in patients with HBV-associated HCC. Upregulation of NEDD4 inhibited proliferation, migration and invasion in HBV-related HCC cell lines. We demonstrated that NEDD4 interacts with HBV X protein (HBx) and that HBx upregulation could reverse the suppression of proliferation and mobility induced by NEDD4 overexpression. Furthermore, we confirmed that NEDD4 induced the degradation of HBx in a ubiquitin/proteasome-dependent manner via K48-linked ubiquitination. Our findings suggest that NEDD4 exerts a tumor-suppressive effect in HBV-associated HCC by acting as an E3 ubiquitin ligase for HBx degradation and provide new insights into the function of NEDD4.

## Introduction

Hepatocellular carcinoma (HCC) is the major subtype of primary liver cancer (1). With the characteristics of insidious onset, rapid progression, and frequent recurrence, HCC ranks third in terms of cancer-related death worldwide (1, 2). Extensive investigations have provided overwhelming evidence that the development of HCC is closely related to metabolic syndrome, alcohol abuse, aflatoxin B1 exposure, and chronic hepatitis B or C virus (HBV or HCV) infection (1). Chronic HBV infection represents the most common pathogenic factor, accounting for up to 54% of HCC cases (3). In the early stage of HBV infection, HBV DNA integrates into the host genome, which results in genomic instability and direct insertional mutagenesis of various oncogenes (4). Persistent expression of HBV regulatory proteins can facilitate the oncogenic transformation of liver cells (3). The HBV X protein (HBx), an HBV-genome-encoded multifunctional regulator, has been proven to be involved in several cellular processes leading to HCC (5). For instance, HBx interferes with the nucleotide excision repair pathway (6); HBx exerts antiapoptotic effects through the PI3K/Akt pathway and p38/MAPK pathway (7, 8); HBx acts as an epigenetic deregulation agent to modulate the transcription of DNA methyltransferase (DNMT) 1 and DNMT3 (9); HBx promotes telomerase activity by increasing the expression of TERT (10). In addition, it has been reported that anti-HBV drugs, such as telbivudine, entecavir, and interferon-α2b, suppress the growth of HBV-related HCC via downregulation of HBx (11).

Ubiquitination, a posttranslational modification, is responsible for numerous complex cellular processes, including protein degradation, protein-protein interactions, and cellular pathway regulation. During the ubiquitination process, ubiquitin (Ub) is covalently attached to lysine residues on the protein substrate through sequential enzymatic reactions accomplished by ubiquitin-activating enzyme E1, ubiquitin-conjugating enzyme E2, and ubiquitin-protein ligase E3 (12). Neural precursor cell expressed developmentally downregulated gene 4 (NEDD4) is one of E3 Ub ligases that has been identified to exert numerous vital cellular processes, such as proteasomal degradation, membrane protein endocytosis, endosomal trafficking and autophagy regulation (13). Recently, NEDD4 has been reported to play either oncogenic or suppressive roles in multiple human cancers. For example, p21 is the substrate of NEDD4 as well as a key regulator of tumor proliferation in colorectal cancer. NEDD4 could target p21 protein for degradation by increasing the ubiquitylation of p21, and promote cell proliferation. N-myc downstream-regulated gene 1 (NDRG1) inhibits colorectal cancer cell proliferation through emulatively antagonizing NEDD4-mediated ubiquitylation of p21, which suggests an oncogenic role of NEDD4 in colorectal cancer (14). In malignant glioma, researchers found that NEDD4 directly promotes cell migration and invasion, but does not affect the proliferation and apoptosis. Besides, NEDD4 could interact with cyclic nucleotide Ras guanine nucleotide exchange facto (CNrasGEF), increase CNrasGEF polyubiquitination and target CNrasGEF for degradation. Downregulated CNrasGEF promotes cell migration and invasion, and facilitates the effect of NEDD4-induced cell motility, which indicates that NEDD4 exerts an oncogenic function in glioma cell motility through ubiquitination of CNrasGEF (15). However, previous study has reported that NEDD4 interacts with Myc oncoproteins, and targets Myc oncoproteins for ubiquitination and degradation by the ubiquitin conjugating enzymes (E2) UbcH5a and UbcH5b. The histone deacetylase Sirtuin 2 (SIRT2) reduces ubiquitin-proteasome pathway-induced N-Myc protein degradation in neuroblastoma and C-Myc protein degradation in pancreatic cancer through repressing NEDD4 gene transcription by directly binding to NEDD4 gene promoter, which contributes to upregulation of Myc oncoproteins and results in cancer cell proliferation (16). From this perspective, NEDD4 exercises a tumor suppressive function. Recent studies have also found that negative regulation of LATS1 and PTEN might be the mechanisms by which NEDD4 promotes HCC progression (17, 18). However, here we described the opposite function of NEDD4 in HBV-associated HCC.

In this study, we first found that HBV-infected HCC patients with high tumor NEDD4 expression experienced superior cumulative survival over those with low tumor NEDD4 expression and that high NEDD4 expression in HBV-positive cell lines inhibited proliferation, migration, and invasion. Second, we identified 245 proteins that may interact with NEDD4 in HepG2.215 cell lines and found that NEDD4 and HBx interact with each other. Finally, our study demonstrated that NEDD4 induced the degradation of HBx in an ubiquitin-proteasome-dependent manner via K48-linked ubiquitination, which suppressed tumor progression in HBV-positive HCC.

## Methods and Materials

### Patients and Specimens

Samples from a total of 199 HCC patients who underwent surgery were obtained from Department of Hepatobiliary Surgery, The Third Xiangya Hospital, Central South University. The clinical characteristics of the patients were collected and are shown in **Supplementary Table S1**. We divided the patients into two groups: HBV-positive patients (N = 104) and HBV-negative patients (N = 95). For RNA extraction, the tissues were immediately stored in liquid nitrogen until further investigation. All experiments were conducted with the approval of the Ethical Committee of The Third Xiangya Hospital, Central South University.

### Cell Culture

HEK293T and HBV-related HCC cell lines, including HepG2.215, HepG3B, SNU182, SNU387, PLC/PRF/5, and MHCC97H, were obtained from the Chinese Academy of Sciences (Shanghai, China). Cells were maintained in Dulbecco’s modified Eagle’s medium (DMEM) (Gibco, USA) supplemented with 10% fetal bovine serum (FBS) (Gibco, USA) and 1% penicillin/streptomycin (Gibco, USA) at 37°C with 5% CO_2._

### Quantitative Reverse Transcription Polymerase Chain Reaction (qRT-PCR)

Total RNA was extracted from liver samples or HCC cells with TRIzol Reagent (Takara, Japan). We then separated and purified total RNA with chloroform, isopropanol, and ethanol. One microgram of RNA was reverse-transcribed into cDNA by the PrimeScript RT reagent Kit (Takara, Japan) according to the manufacturer’s instructions, and qRT-PCR analysis was performed using SYBR® Green Master Mix (Takara, Japan). The relative expression of target genes was analyzed and shown as the fold change (2-ΔΔCt). The primer sequences of the target genes were as follows: NEDD4, 5′-GGAGTTGCCAGAGAATGGTT-3′ (forward); 5′-TTGCCATGATAAACTGCCAT-3′ (reverse). HBX, 5’- TGTCAACAACCGACCTTGAG-3’ (forward); 5’-AAAGTTGCATGGTGCTGGTG-3’ (reverse). GAPDH, 5’-GGACCTGACCTGCCGTCTAG-3’ (forward); 5’-GTAGCCCAGGATGCCCTTGA-3’ (reverse).

### Plasmid Construction and Transfection

Expression plasmid constructs, including full-length pcDNA3.1(+)-Flag-HBx, full-length pcDNA3.1(+)-Myc-NEDD4 (*Homo sapiens*), pcDNA3.1(+)-Myc-NEDD4 catalytic inactive mutant (cysteine 1197 to alanine, *Homo sapiens*), full-length pcDNA3.1(+)-HA-Ubiquitin (ubiquitin B, UBB, *Homo sapiens*), full-length pcDNA3.1(+)-HA-K48-Ubiquitin (ubiquitin B, UBB, *Homo sapiens*), pcDNA3.1(+)-HA-K63-Ubiquitin (ubiquitin B, UBB, *Homo sapiens*) were all constructed by and purchased from Obio Technology (Shanghai) Corp, Ltd. NEDD4 small-interfering RNA (siRNA) was designed and synthesized by GenePharma (Shanghai, China). A Lipofectamine 3000 Transfection Kit (Invitrogen, L3000-015) was used for transfection. Transfection was performed according to the manufacturer’s instructions with minor modifications. Briefly, 293T cells were seeded in 6-well plates at a density of 3 × 10^5^ cells/well. The use of each plasmid was 2.5 µg/well with 5 µl Lipo3000 and 5 µl P3000 according to the manufacturer’s instructions. The total amount of transfected plasmids in each well was equalized by adding empty pcDNA3.1(+)-vector.

### MTT Assay

We used a 3-(4,5-dimethylthiazol-2-yl)-2,5-diphenyltetrazolium bromide (MTT) kit (Abcam, ab211091) to evaluate cell viability. Cells were inoculated in 96-well plates (1,500 cells per well) for 48 h, and then 20 μl of MTT solution was added to each well and incubated at 37°C for 4 h. After removing the supernatants, 150 μl of DMSO was added to each well, and the absorbance was measured at 570 nm with an automated microplate reader (Thermo Fisher Scientific, USA).

### EdU Proliferation Assay

HepG2.215 cells were seeded into 24-well plates and treated for 48 h. Subsequently, according to the instructions of BeyoClick™ EdU Cell Proliferation Kit with Alexa Fluor 488 (Beyotime, Jiangsu, China), EdU solution was added into each well and the cells were incubated for a further 2 h. Then, the cells were washed by PBS and fixed with 4% paraformaldehyde for 20 min. After that, 0.5% Triton X-100 was added to increase the permeability of the cells. Subsequently, the cells were incubated with Click Reaction Mixture for 30 min and stained with Hoechst (1:1000) for 2 min. Finally, the cells were observed using a fluorescence microscope. Edu positive cell rate = number of green fluorescence-labeled cells/number of blue fluorescence-labeled cells × 100%.

### Transwell Assay

We used the serum-free DMEM cell suspension at a density of 1 × 10^5^/ml.

Fibronectin and BD™ Matrigel (BD, USA) were precoated on Transwell inserts for the migration assays and invasion assays, respectively.

We added 100 μl of the cell suspension to the upper chamber of the Transwell inserts and 600 μl of DMEM with 10% FBS to the lower chamber. Cells were incubated at 37°C for 24 h for the migration assay and 48 h for the invasion assay. Then, we fixed the cells on the lower insert surface with 4% paraformaldehyde and stained them with 1% crystal violet. Six different microscopic fields of three independent inserts were captured to count the cells.

### Western Blotting

Cells were harvested and lysed in RIPA buffer containing protease and phosphatase inhibitors for 30 min on ice. After centrifugating lysates at 14,000 rpm at 4°C for 15 min, we collected the supernatants and determined protein concentrations using the BCA protein assay (Thermo Scientific). Equal amounts of each sample diluted in 5x SDS loading buffer were subjected to SDS-polyacrylamide gel electrophoresis and then transferred to polyvinylidene fluoride (PVDF) membranes (Millipore) for 2 h. Five percent nonfat dry milk dissolved in TBST (150 mM NaCl, 50 mM Tris-HCl, pH 7.5, and 0.05% Tween-20) was used to block the membranes for 2 h, and then the membranes were incubated with primary antibodies overnight at 4°C. The primary antibodies used in this study were GAPDH (1:1000, Abcam, ab8245), NEDD4 (1:1000, Cell Signaling Technology, 2740), HBX (1:1000, Abcam, ab235), and Ki-67 (1:1000, Abcam, ab16667). Then, we washed the membranes three times in TBST and incubated the membranes with the appropriate HRP-conjugated secondary antibodies (1:3000, Beyotime Institute of Biotechnology, A0216, A0208, A0192) for 1.5 h at room temperature. Then, the membranes were washed an additional three times with TBST. The bands on membranes were visualized using an ECL western blotting kit (Millipore).

### Coimmunoprecipitation and LC-MS/MS

HepG2.215 cell lines were harvested on ice in modified RIPA buffer containing 50 mM Tris•HCl (pH 7.5), 150 mM NaCl, 0.1% (vol/vol) Triton X-100, 0.5% (wt/vol) sodium deoxycholate, 0.1% (wt/vol) SDS, 1 mM EDTA, 50 mM N-ethylmaleimide, 1 mM NaF, 1 mM Na3VO4, 1 mM PMSF, and 1 μg/ml each of aprotinin, leupeptin, and pepstatin. The cell lysates (approximately 400 μg of total protein) were incubated with an antibody against NEDD4 (4 µl, Cell Signaling Technology, 3607), HBX (4 µl, Abcam, ab2741), Flag-Tag (4 µl, Cell Signaling Technology, 14793) or their IgG control (Cell Signaling Technology, 3452 or 37988) at 4°C overnight. Then, protein-G agarose beads (40 L, Beyotime Biotechnology) were added, and the mixture was incubated at 4°C for another 3 h. The agarose beads were collected, washed, and resuspended in 60 μl of sample buffer containing 50 mM Tris•HCl, pH 7.6, 2% (wt/vol) SDS, 10% (vol/vol) glycerol, 10 mM DTT, and 0.2% bromophenol blue. Afterward, the samples were boiled for 10 min. Liquid chromatography with tandem mass spectrometry (LC-MS/MS) was used to analyze the interacting proteins of NEDD4 in HepG2.215 cell lines. The entire LC-MS/MS procedure was performed by Applied Protein Technology (ATP) Company. The western blotting protocols were reported above. A special secondary antibody (1:1000, Abcam, ab131366), which only recognizes native (nonreduced) antibodies to highly minimize the detection of heavy and light chains, was used to test the IP samples to avoid the influence of the IP antibodies in the IP samples.

### Immunofluorescence Staining

HepG2.215 cell lines were seeded on sterile glass coverslips. When cells reached approximately 60% confluence, the growth medium was aspirated. Cells were washed with ice-cold PBS three times before fixing in 4% paraformaldehyde for 30 min. To permeabilize the cells, 0.1% Triton X-100 was added for 15 min at room temperature, and 5% normal goat serum was used to block cells for 30 min. A primary antibody against NEDD4 (1:100, Cell Signaling Technology, 2740) and HBX (1:100, Abcam, ab235) was added, and the cells were incubated overnight at 4°C. After the cells were washed with PBS three times, anti-rabbit IgG (1:500, Cell Signaling Technology, 4413) and anti-mouse IgG (1:500, Cell Signaling Technology, 4408) were added, and the cells were incubated for another 1 h at room temperature. 4’,6-diamidino-2-phenylindole (DAPI) was used to counterstain the nuclei. Thereafter, the coverslips were mounted on glass slides with antifade mounting medium (Beyotime, P0126). Thereafter, the samples were viewed under a laser scanning confocal microscope at different wavelengths of 488 nm, 555 nm, and 405 nm. Images were observed under a confocal microscope (Carl Zeiss LSM710, Jena, Germany).

### Flag-HBX Degradation Assay

HepG2.215 cell lines were seeded in 6-well plates at a density of 3 × 105 cells/well and transfected with Flag-HBX+empty vector plasmids or Flag-HBX+Myc-NEDD4 plasmids for 72 h. Protein lysates were prepared at the indicated time points after the addition of cycloheximide (CHX) (10 μM). Equal amounts of protein were separated by SDS-PAGE. The levels of Flag-HBX were determined by immunoblotting and quantified at the indicated time points. HepG2.215 cells were seeded in 6-well plates at a density of 3 × 105 cells/well and transfected with Flag-HBX and Myc-NEDD4 plasmids for 72 h. Cells were treated with chloroquine (CQ, 50 µM, Cell Signaling Technology, 14774) or MG132 (20 µM, Selleck, S2619) for another 12 h to block the autophagy-lysosome or ubiquitin-proteasome pathway, respectively. Protein lysates were harvested after that, and the protein level of Flag-HBX was evaluated by western blotting.

### *In Vivo* Ubiquitination Assay

To prepare cell lysates, HepG2.215 cell lines were solubilized in ice-cold modified lysis buffer (50 mM Tris, pH 7.4, 150 mM NaCl, 10% glycerol, 1 mM EDTA, 1 mM EGTA, 1% SDS, 1 mM Na3VO4, 1 mM DTT and 10 mM NaF) supplemented with a protease inhibitor cocktail after transfection with different combinations of plasmids for 72 h. The cell lysate was incubated at 60°C for 10 min. The lysate was then diluted 10 times with modified lysis buffer without SDS. The lysate was incubated with Flag-Tag primary antibody (1:100, Cell Signaling Technology, 14793) for 3 h at 4°C. Protein-G agarose beads (40 L, Beyotime Biotechnology) were added, and the lysate was rotated gently for 8 h at 4°C. The immunoprecipitates were washed at least three times in wash buffer (50 mM Tris, pH 7.4, 150 mM NaCl, 10% glycerol, 1 mM EDTA, 1 mM EGTA, 0.1% SDS, 1 mM DTT and 10 mM NaF). Proteins were recovered by boiling the beads in 5X SDS sample buffer and analyzed by western blotting using the HA-Tag primary antibody (1:1000, Cell Signaling Technology, 3724).

### *In Vivo* Experiments

We used HepG2.215 cells and NEDD4-overexpressing HepG2.215 cells to further verify the function of NEDD4 *in vivo*. Cells suspensions at a concentration of 1 × 10^7^ cells per 100 μl serum-free medium were prepared. *In vivo* experiments were performed in the Animal Laboratory Center, and ethics approval was obtained from the Committee for Experimental Animal Studies of Central South University. Four- to six-week-old male nude (BALB/c nu/nu) mice were subcutaneously injected with 100 μl of cell suspension in the left armpits. Tumor volumes were measured and recorded each week. The mice were sacrificed one month after injection. The tumors were excised, photographed, and weighed.

### Immunohistochemistry

Xenograft tumor tissues were fixed in formalin for 48 h and were prepared into paraformaldehyde-fixed, paraffin-embedded sections. The sections were deparaffinized in xylene, rehydrated in ethanol and rinsed in PBS before being treated with TE (10 mM Tris/1 mM EDTA, [pH 9.0]). Then, sections were incubated with 3% hydrogen peroxide. After blocking with 200 μl of normal goat serum (ZSGB-BIO, China) for 1 h at 37°C, sections were incubated with 200 μl Ki-67 primary antibody (1:200, Abcam, ab16667) overnight at 4°C. Then, the sections were washed with PBS three times and incubated with diluted streptavidin-peroxidase HRP conjugates. After that, immunohistochemical staining was performed with hematoxylin before analysis under a microscope. The numbers of Ki-67 positive cells were counted in three random fields of view per slide, and the percentage of Ki-67 positive cells was calculated.

### Statistical Analysis

All results were determined from three independent experiments under the same conditions. All data are expressed as the mean ± standard deviation (SD). Differences among groups were compared by Student’s t-tests and one-way ANOVA. The Kaplan-Meier test was used to evaluate overall survival (OS) and progression-free survival (PFS). Differences were considered statistically significant when p values were less than 0.05 (p < 0.05).

## Results

### NEDD4 Overexpression Is Associated With Increased OS and PFS in HBV-Positive HCC Patients

**Figure 1:**
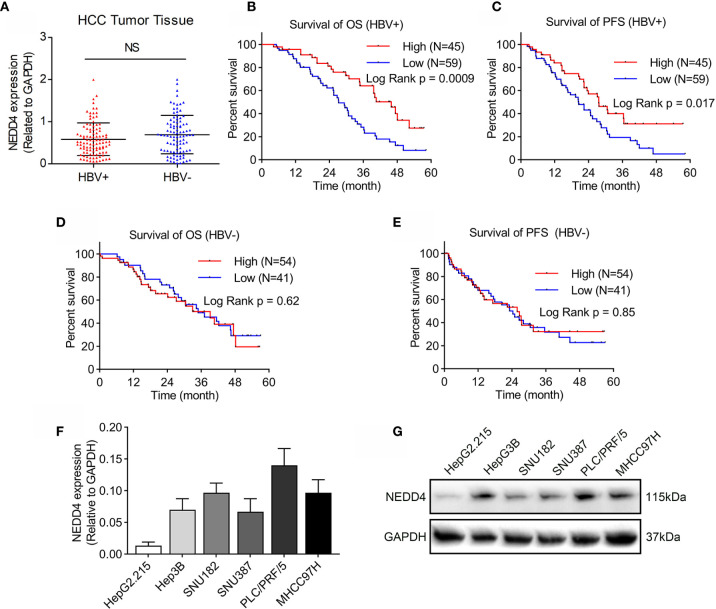
NEDD4 expression in HCC tissues and cell lines. **(A)** NEDD4 expression in HCC specimens with or without HBV infection was evaluated through qRT-PCR. There was no correlation between NEDD4 expression and HBV exposure in HCC tissue. **(B)** and **(C)** High NEDD4 expression was related to better OS and PFS than low NEDD4 expression in patients with HBV-associated HCC. **(D)** NEDD4 expression was unrelated to OS for patients with HBV-negative HCC. **(E)** NEDD4 expression was unrelated to PFS for patients with HBV-negative HCC. **(F)** NEDD4 mRNA expression in HBV-associated cell lines. **(G)** NEDD4 protein expression in HBV-associated cell lines. (Data are presented as the mean ± SD. NS, no significance (p < 0.05).

Previous studies have revealed that the role of NEDD4 in human cancers remains controversial. Although it has been proven that NEDD4 acts as a proto-oncogene in HCC, studies have not made a distinction between HBV-positive and HBV-negative HCC. Therefore, we first investigated the correlation between NEDD4 expression and HBV exposure through analyzing tumor samples from 104 patients with HBV-positive HCC and 95 patients with HBV-negative HCC using qRT-PCR. We found that there was no significant difference in NEDD4 expression between HBV-positive and -negative HCC **(** **Figure 1A** **)**. However, Kaplan-Meier analysis revealed that HBV-positive HCC patients with high NEDD4 expression had significantly longer OS and PFS times (p = 0.0009 and p = 0.017, **Figures 1B, C** **)**. There was no relationship between NEDD4 expression and OS and PFS for patients with HBV-negative HCC **(** **Figures 1D, E** **)**. These results indicated that NEDD4 might play a tumor-suppressive role in HBV-associated HCC.

### NEDD4 Overexpression Inhibits Proliferation and Mobility in HBV-Associated HCC Cell Lines

**Figure 2:**
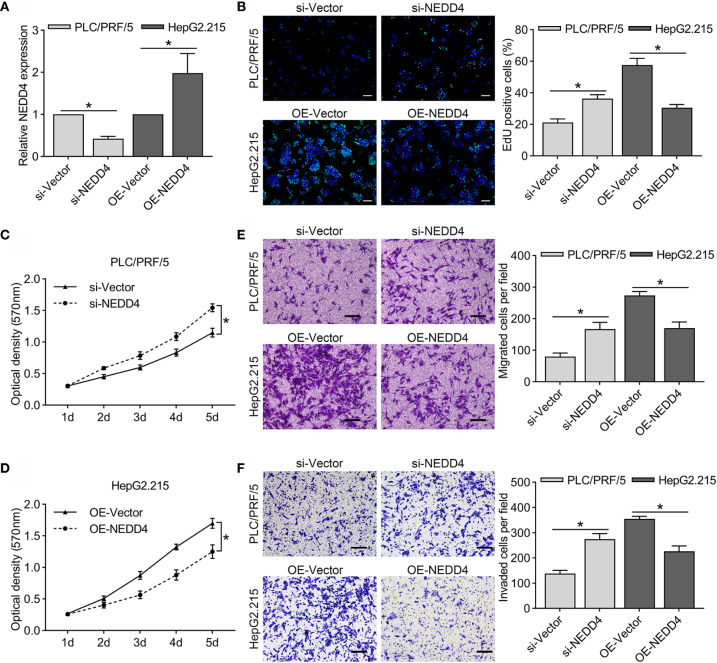
Regulatory effect of NEDD4 in HBV-associated HCC cell lines. **(A)** NEDD4 mRNA expression in NEDD4 knockdown PLC/PRF/5 cells and NEDD4 overexpression HepG2.215 cells as well as their negative controls. **(B)** Edu assay was performed to compare the cell growth ability in NEDD4 knockdown PLC/PRF/5 cells and NEDD4 overexpression HepG2.215 cells with their negative controls (Green fluorescence: EdU, Blue fluorescence: Hoechst). **(C)** Downregulated NEDD4 in PLC/PRF/5 cells promoted proliferation. **(D)** NEDD4 overexpression in HepG2.215 cells inhibited proliferation. **(E)** Downregulated NEDD4 promoted migration in PLC/PRF/5 cells, while upregulated NEDD4 inhibited migration in HepG2.215 cells. **(F)** NEDD4 depletion promoted invasion in PLC/PRF/5 cells, whereas NEDD4 overexpression inhibited invasion in HepG2.215 cells. Data are presented as the mean ± SD. *p < 0.05. (si-NEDD4 represents NEDD4 knockdown PLC/PRF/5 cells, and si-Vector represents the negative control; OE-NEDD4 represents NEDD4 overexpression HepG2.215 cells, and OE-Vector represents the negative control) (n = 3 independently replicated experiments; white scale bars: 100 μm, black scale bars: 50 μm).

The role of NEDD4 in HBV-associated HCC remains unclear. Therefore, we first detected basic NEDD4 expression levels in HBV-associated HCC cell lines, including HepG2.215, HepG3B, SNU182, SNU387, PLC/PRF/5, and MHCC97H **(** **Figures 1F, G** **)**. Next, we selected PLC/PRF/5 cells (high NEDD4 expression) and HepG2.215 cells (low NEDD4 expression) to investigate the regulatory effect of NEDD4 in HBV-associated HCC. We generated NEDD4 knockdown PLC/PRF/5 cells and NEDD4 overexpression HepG2.215 cells (si-NEDD4 and OE-NEDD4) as well as their negative controls (si-Vector; OE-Vector). The expression of NEDD4 in these cells was identified at the mRNA level **(** **Figure 2A** **).** The EdU assays were performed to determine the regulatory effect of NEDD4 on proliferation. The results demonstrated that si-NEDD4 significantly increase the ratio of proliferating cells to 36.3% compared with the control (21.1%) in PLC/PRF/5 cells. Conversely, over-expression of NEDD4 markedly decreased the proliferation ability in HepG2.215 cells **(** **Figure 2B** **)**. In addition, the MTT assay implied that NEDD4 knockdown promoted the proliferation of HBV-associated HCC cells, while NEDD4 overexpression inhibited their growth in a time-dependent manner **(** **Figures 2C, D** **)**.

We further conducted a Transwell assay to detect cell migration and invasion *in vitro*. We found that the expression of NEDD4 inhibited both migration and invasion in HBV-associated HCC cells **(** **Figures 2E, F** **)**. Surprisingly, our results were completely opposite to those of previous studies showing that NEDD4 promotes the proliferation and mobility of HBV-negative HCC cells, including SMMC-7721, QGY-7703, and Huh-7 cells (17, 18). These results suggested that molecular interactions between NEDD4 and HBV-associated proteins might exist.

### NEDD4 Interacts With HBx in HBV-Associated HCC Cell Lines

**Figure 3:**
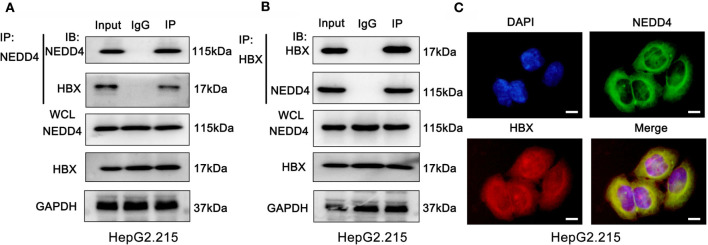
NEDD4 interacts with HBx in HBV-associated hepatocellular cells. **(A)** and **(B)** Coimmunoprecipitation was conducted with NEDD4 and HBx antibodies. The coimmunoprecipitated mixture was separated by SDS-PAGE and evaluated by immunoblotting. NEDD4 and HBx interact with each other in HepG2.215 cells. **(C)** Immunofluorescence assay demonstrated the colocalization of NEDD4 and HBx in HepG2.215 cells (green NEDD4, red HBx, blue, DAPI) (n = 3 independently replicated experiments; white scale bars: 10 μm). IP, Immunoprecipitation; IB, immunoblot; WCL, whole cells lysate.

To validate the interplay of NEDD4 and proteins relative to HBV infection, we performed coimmunoprecipitation (Co-IP) and LC-MS/MS experiments in HBV-associated hepatocellular cells. We identified 245 proteins that may interact with NEDD4 in HepG2.215 cell lines **(** **Supplementary document 1** **)**. Surprisingly, we noticed that HBx was involved in the Co-IP complex **(** **Supplementary document 1** **)**. HBx is a 17-kDa transcriptional coactivator produced by HBV virus that plays a significant role in HBV-associated hepatocarcinogenesis. Therefore, we further conducted reciprocal Co-IP/western blot assays to confirm again that endogenous NEDD4 and HBx interact with each other in the HepG2.215 cell line **(** **Figures 3A, B** **)**. Moreover, immunofluorescence assays showed NEDD4 and HBx colocalization in the HepG2.215 cell line **(** **Figure 3C** **)**.

### NEDD4 Diminishes the Expression of HBx at the Protein Level but Not at the mRNA Level

**Figure 4:**
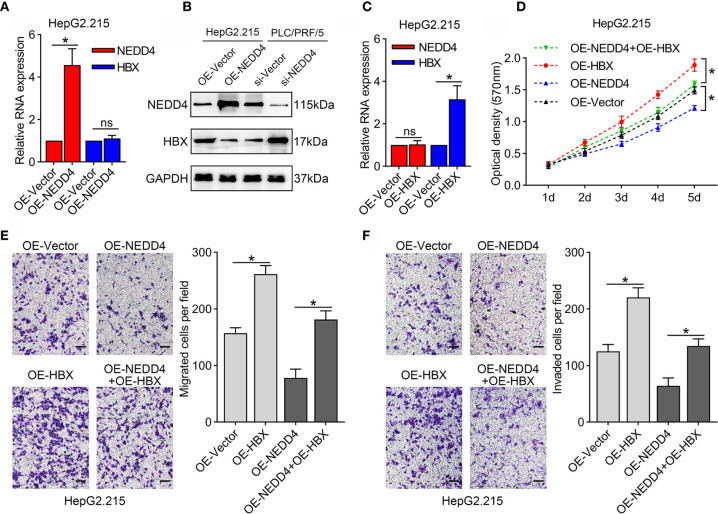
HBx upregulation reverses the suppression of proliferation and mobility induced by NEDD4 overexpression in HBV-associated HCC cells in HepG2.215 cell line. **(A)** NEDD4 upregulation did not affect the mRNA level of HBx. **(B)** The protein level of HBx was downregulated after NEDD4 overexpressed. **(C)** HBx mRNA expression in HBx-overexpressing cells, NEDD4-overexpressing cells, cells overexpressing both HBx and NEDD4, and negative control cells. **(D)** Upon upregulation of HBx, NEDD4-induced proliferation suppression was recovered. **(E)** HBx upregulation compensated for NEDD4-induced migration inhibition. **(F)** NEDD4-induced invasion suppression was reversed after HBx upregulation. Data are presented as the mean ± SD. *p < 0.05, NS, no significance (p > 0.05). (OE-HBx+OE-NEDD4 represents upregulated HBx in NEDD4 overexpression HepG2.215 cells; OE-HBx represents HBx overexpression HepG2.215 cells; OE-NEDD4 represents NEDD4 overexpression HepG2.215 cells and OE-Vector represents the negative control) (n = 3 independently replicated experiments; scale bars: 50 μm).

As mentioned above, NEDD4 mostly acts as an E3 ubiquitin ligase that interacts with other proteins to play a role in certain physiological and pathological conditions. Therefore, we conducted qRT-PCR and western blotting assays to investigate HBx expression in NEDD4-overexpressing cells. The results showed that NEDD4 upregulation did not affect the mRNA level of HBx **(** **Figure 4A** **)**. However, the protein level of HBx was downregulated after NEDD4 overexpressed **(** **Figure 4B** **)**. These results indicated that NEDD4 negatively regulates HBx in the process of posttranslational modification.

### HBx Overexpression Reverses the Suppression of Proliferation and Mobility Induced by NEDD4 Overexpression in HBV-Associated HCC Cells

To determine whether the interplay of NEDD4 and HBx attenuates HBV-associated HCC progression, we upregulated HBx expression in NEDD4-overexpressing cells (OE-HBx+OE-NEDD4) and generated HBx-overexpressing cells (OE-HBx) as a control group **(** **Figure 4C** **)**. Our results showed that upon upregulation of HBx, NEDD4-induced proliferation suppression was recovered **(** **Figure 4D** **)**. Similar results were also found for cell metastatic capacity. HBx upregulation compensated for NEDD4-induced migration and invasion inhibition **(** **Figures 4E, F** **)**. These results suggested that NEDD4 might attenuate the expression of HBx protein to suppress HBV-associated HCC progression.

### NEDD4 Induces the Degradation of HBx in a Ubiquitin/Proteasome-Dependent Manner *via* K48-Linked Ubiquitination

**Figure 5:**
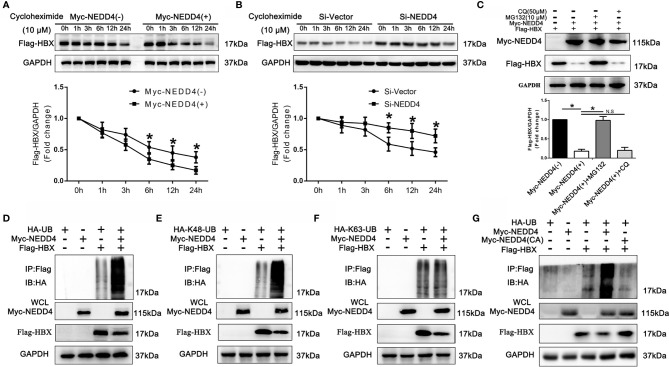
NEDD4 induced the degradation of HBx in a ubiquitin/proteasome-dependent manner via K48-linked ubiquitination. **(A)** Flag-HBx was transfected into 293T cells with Myc-NEDD4 or empty vectors. After transfection of plasmids for 48 h, protein lysates were prepared at the indicated time points after treatment with 10 μM cycloheximide, and the Flag-HBx protein levels were evaluated by western blotting. The results demonstrated that the Flag-HBx degradation rate was significantly higher when HBx was coexpressed with Myc-NEDD4. **(B)** Flag-HBx was transfected into 293T cells with Si-Vector or Si-NEDD4. After transfection for 48 h, protein lysates were prepared at the indicated time points after treatment with 10 μM cycloheximide, and the Flag-HBx protein levels were evaluated by western blotting. The results demonstrated that the Flag-HBx degradation rate was significantly lower with NEDD4 knockdown. **(C)** Flag-HBx and Myc-NEDD4 expression vectors were cotransfected into 293T cells for 48 h, after which 293T cells were treated with chloroquine (CQ, 50 µM) or MG132 (10 µM) for another 12 h. Western blotting demonstrated that MG132 reversed the degradation of Flag-HBx induced by Myc-NEDD4, while CQ had little effect. **(D)** Flag-HBx and HA-Ubiquitin was transfected into 293T cells with Myc-NEDD4 or empty vectors. After transfection of plasmids for 48 h, protein lysates were harvested and Flag-HBx was immunoprecipitated with anti-Flag antibody. Western blotting demonstrated that Myc-NEDD4 significantly induced the ubiquitination of Flag-HBx. **(E)** Flag-HBx and HA-K48-Ubiquitin was transfected into 293T cells with Myc-NEDD4 or empty vectors. After transfection of plasmids for 48 h, protein lysates were harvested and Flag-HBx was immunoprecipitated with anti-Flag antibody. Western blotting demonstrated that Myc-NEDD4 significantly induced the K48-linked ubiquitination of Flag-HBx. **(F)** Flag-HBx and HA-K63 -Ubiquitin was transfected into 293T cells with Myc-NEDD4 or empty vectors. After transfection of plasmids for 48 h, protein lysates were harvested and Flag-HBx was immunoprecipitated with anti-Flag antibody. Western blotting demonstrated that Myc-NEDD4 did not induce the K63-linked ubiquitination of Flag-HBx. **(G)** Flag-HBx and HA-Ubiquitin was transfected into 293T cells with Myc-NEDD4 or Myc-NEDD4 catalytically inactive mutant (cysteine 1197 to alanine). After transfection of plasmids for 48 h, protein lysates were harvested and Flag-HBx was immunoprecipitated with anti-Flag antibody. Western blotting demonstrated that Myc-NEDD4 catalytically inactive mutant did not induce the ubiquitination of Flag-HBx. Data in A and B are presented as the mean ± SD. *p < 0.05, NS, no significance (p > 0.05). (n = 3 independently replicated experiments). HA-UB, full-length pcDNA3.1(+)-HA tagged-Ubiquitin (ubiquitin B, UBB, *Homo sapiens*); HA-K48-UB, full-length pcDNA3.1(+)-HA tagged-Lys48 Ubiquitin (ubiquitin B, UBB, *Homo sapiens*); HA-K63-UB, pcDNA3.1(+)-HA tagged-Lys63-Ubiquitin (ubiquitin B, UBB, *Homo sapiens*).

Since NEDD4 suppresses the expression of HBx at the protein level and mostly acts as an E3 ubiquitin ligase, we speculated that NEDD4 might affect the degradation of HBx protein. We then evaluated the degradation kinetics of Flag-HBx coexpressed with Myc-NEDD4 or NEDD4 knockdown in 293T cells, and we demonstrated that the Flag-HBx degradation rate was significantly higher when coexpressed with Myc-NEDD4 while significantly lower when knocking down NEDD4 simultaneously **(** **Figures 5A, B** **)**. The degradation of proteins occurs mainly through the autophagy-lysosome or ubiquitin-proteasome pathway. Thus, we added chloroquine (CQ, 50 µM) or MG132 (10 µM) to block the autophagy-lysosome pathway or ubiquitin-proteasome pathway, respectively. We demonstrated that MG132 reversed the degradation of Flag-HBx mediated by Myc-NEDD4, while CQ had little effect **(** **Figure 5C** **)**. This result indicated that NEDD4 degraded HBx through the ubiquitin-proteasome pathway instead of the autophagy-lysosome pathway. We further coexpressed HA-ubiquitin, Myc-NEDD4 and Flag-HBx in 293T cells and found that Myc-NEDD4 significantly induced the ubiquitination of Flag-HBx **(** **Figure 5D** **),** and the type of HBx ubiquitination modulated by NEDD4 was K48-linked instead of K63-linked **(** **Figures 5E, F** **).** Finally, we constructed a plasmid with a catalytically inactive mutant NEDD4 (cysteine 1197 to alanine). The results demonstrated that the catalytically inactive NEDD4 mutant did not induce the ubiquitination of HBx (**Figure 5G**). In summary, NEDD4 might act as an E3 ubiquitin ligase to ubiquitinate HBx through the K48 ubiquitin chain and thus degrade HBx.

### NEDD4 Overexpression Inhibits HBV-Associated HCC Progression *In Vivo*

**Figure 6:**
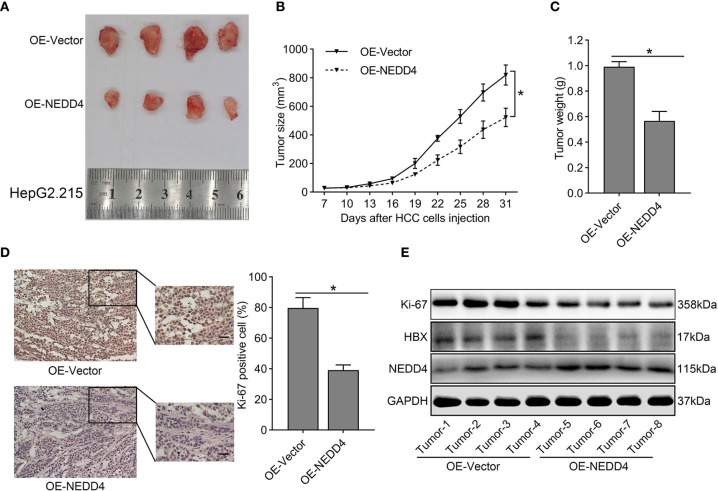
The tumor suppressor role of NEDD4 in HBV-associated HCC *in vivo*. **(A)** A representative photograph of xenograft tumors from mice injected with NEDD4-overexpressing HepG2.215 cells and negative control cells. **(B)** A tumor size curve shows the volume variation of the xenografts from mice injected with NEDD4-overexpressing HepG2.215 cells and control cells. **(C)** Weight of the tumors from mice injected with NEDD4-overexpressing HepG2.215 cells and control cells were measured after the mice were killed. **(D)** Representative images of immunohistochemical staining patterns and western blotting bands for Ki-67 in xenograft tumors in the NEDD4 overexpression group and control group. **(E)** Western blot results showed that over-expression of NEDD4 could markedly inhibit the expression of HBX and Ki-67 in xenograft tumors. Data are presented as the mean ± SD. *p < 0.05 (OE-NEDD4 represents the NEDD4 overexpression group, and OE-Vector represents the control group.) (n = 3 independently replicated experiments; scale bars: 50 μm).

To verify whether NEDD4 upregulation could attenuate the malignant features of HBV-associated HCC *in vivo*, xenograft tumor models were established in nude mice. We noticed that NEDD4 overexpression sharply decreased tumor size compared to that in the control group **(** **Figure 6A** **)**. Both tumor volume and tumor weight in the OE-NEDD4 group were significantly smaller than those of the control group **(** **Figures 6B, C** **)**. Subsequently, we measured the expression of Ki-67 (a protein related to proliferation) in xenograft tumors through immunohistochemistry and western blotting. We found that the expression of Ki-67 was diminished in NEDD4-overexpressing xenograft tumors compared to control tumors **(** **Figures 6D, E** **)**. Taken together, these results further confirmed that NEDD4 upregulation inhibited HBV-associated HCC progression.

## Discussion

Here, we used HBV-associated HCC cells to further explore the potential function of NEDD4 in the context of HBV infection. We have clearly described that NEDD4 contributes to inhibiting HBV-associated HCC progression via the NEDD4-HBx interaction, which results from the K48-linked ubiquitination and degradation of HBx by NEDD4.

NEDD4 is frequently overexpressed in many different human malignancies and can act as either an oncogene or a tumor suppressor (19). Recently, several studies revealed that NEDD4 is highly expressed in HCC and participates in HCC progression (17, 18). However, few studies have focused on the correlation between NEDD4 and HBV-associated HCC. In this study, we compared NEDD4 expression between HBV-positive and HBV-negative HCC tumor tissues. Although there was no correlation between NEDD4 expression and HBV exposure, we noticed that HBV-associated HCC patients with high NEDD4 expression had better survival than those with low NEDD4 expression. This result was quite different from that of Xiaofeng Hang et al., who analyzed 219 HCC patients and demonstrated that NEDD4 overexpression is associated with decreased overall survival (18). Therefore, our focus was to investigate whether the function of NEDD4 was different in HBV-associated HCC. Our findings indicated that NEDD4 upregulation in HBV-associated HCC cell lines inhibited proliferation, migration, and invasion. Although these results were completely opposite to those of previous studies using HBV-negative cell lines, they were not incomprehensible because the regulatory effect of NEDD4 in cancer remains elusive. It has been reported that NEDD4 could negatively regulate the tumor suppressor PTEN via polyubiquitination for degradation or positively modulate PTEN nuclear transport through monoubiquitination (20, 21). However, there may be no correlations between NEDD4 and PTEN (22, 23). In breast cancer, Jia Liu et al. found that depletion of β-TRCP or inactivation of CKIδ increased NEDD4 abundance, leading to the downregulation of PTEN, which activated the oncogenic mTOR/Akt pathway and promoted cell proliferation (24). In contrast, NEDD4 reduced the PIP5Kα-dependent PIP2 pool to inhibit breast cancer cell proliferation through the PI3K/Akt pathway (25). NEDD4 downregulation in breast cancer cells elevated HER3 expression and enhance AKT and ERK signaling, resulting in cell proliferation and invasion (26). In gastric carcinoma, the role of NEDD4 is equally controversial. Kim et al. found that NEDD4 was expressed in the majority of gastric cancer tissue, while Zhen Yang et al. observed no significant difference in NEDD4 expression between primary gastric tumors and tumor adjacent tissues (23, 27). The expression level of NEDD4 was decreased from gastric dysplasia to gastric carcinoma (23). Based on the ambiguous function of NEDD4 in human cancers and our discrepant discovery in HBV-associated HCC, we speculated that NEDD4 might participate in favorable regulation during the process of hepatocarcinogenesis induced by HBV infection.

To further explore the mechanism of NEDD4 in HBV-associated HCC, we screened 245 proteins interacting with NEDD4 and noticed that HBx was a downstream target in this process. HBx is one of the proteins encoded by the HBV genome, which participates in viral replication and infection, the transactivation of cellular promoters and enhancers through protein-protein interaction, and the regulation of host cellular genes and signal transduction cascades (5). There is growing evidence that Hbx is responsible for the pathogenesis of HBV-related liver diseases, and downregulation of HBx is one of the therapeutic mechanisms for HBV-related HCC (11). In this study, we observed that NEDD4 interacted with HBx and negatively controlled the protein expression of HBx. HBx upregulation could reverse the suppression of proliferation and mobility induced by NEDD4 overexpression. These findings indicate that the different regulatory effects of NEDD4 in HBV-associated HCC cells might be attributed to the NEDD4-HBx interaction.

Thus, the question remains regarding how NEDD4 regulates HBx. NEDD4 is a member of the E3 ubiquitin ligase family and exerts its functions to some extent through degradative ubiquitination of its downstream substrates in both physiological and pathological conditions (13, 19–21, 28). However, only a few previous studies have concentrated on the role of the E3 ubiquitin ligase in HCC. Susie A. Lee et al. found that upregulated NEDD4 was correlated with low Spry2 protein levels in HCC and confirmed that depletion of NEDD4 decreased ubiquitinated levels of Spry2, which suggested a possible role of NEDD4 in Spry2 degradation (29). Another study indicated that NEDD4 regulated the degradation of GUCD1, whose function is to regulate normal and abnormal cell growth in the liver, through the ubiquitin-proteasome system (30). Neither study directly explained the association between NEDD4-induced degradative ubiquitination and hepatocarcinogenesis. On the other hand, previous studies demonstrated that HBx could be recognized as a substrate by E3 ubiquitin ligases for degradation (31). For example, TRIM5γ could recruit TRIM31 and form a TRIM5γ-TRIM31-HBx complex that promotes proteasomal degradation of HBx (32). These findings indicated that Hbx may be a downstream substrate of NEDD4 and regulated through ubiquitination-dependent degradation. In addition, protein degradation can be mediated by the ubiquitin-proteasome pathway or the autophagy-lysosome pathway (33, 34). The type of ubiquitin chain linkage determines the fate of the target substrate. Lys48-linked polyubiquitin chains often label proteins for degradation, while K63-linked ubiquitination usually regulates protein functions or degradation through the lysosomal pathway (28). In this study, for the first time, we verified that NEDD4 promotes the degradation of HBx. We used chloroquine or MG132 to block the autophagy-lysosome pathway or ubiquitin-proteasome pathway, respectively, and confirmed that NEDD4 degrades HBx through the ubiquitin-proteasome pathway. Our in-depth analysis indicated that NEDD4 ubiquitinates and degrades HBx via a K48 ubiquitin chain. These findings suggested that Hbx may be an unveiled substrate of NEDD4 and could explain the discrepant function of NEDD4 in HBV-associated HCC.

In summary, our study first demonstrated that NEDD4 expression was irrelevant to HBV exposure in HCC tumor tissue but that high NEDD4 expression was related to a better OS and PFS than low expression in patients with HBV-associated HCC. Second, we found that NEDD4 inhibited proliferation, migration, and invasion in HBV-related HCC cell lines. Moreover, we identified that NEDD4 interacts with HBx and negatively controls HBx expression at the protein level but not at the mRNA level. HBx upregulation reversed the suppression of proliferation and mobility induced by NEDD4 overexpression. Furthermore, we confirmed that NEDD4 induced the degradation of HBx in a ubiquitin/proteasome-dependent manner via K48-linked ubiquitination. Finally, the tumor suppressor role of NEDD4 in HBV-associated HCC was observed *in vivo*. Our research provides new insight into the function of NEDD4 in HCC. However, there are still some limitations to this study. First, evaluating the upstream and/or downstream signal transduction pathways associated with the NEDD4-HBx complex is of great importance. Second, the discrepant role of NEDD4 in human cancers is attributed to the dual regulation of PTEN, but the regulatory effect of NEDD4 on PTEN in HBV-associated HCC remains unclear. These limitations will be the focus of our future study.

## Data Availability Statement

The raw data supporting the conclusions of this article will be made available by the authors, without undue reservation.

## Ethics Statement

The studies involving human participants were reviewed and approved by the Ethical Committee of The Third Xiangya Hospital, Central South University. The patients/participants provided their written informed consent to participate in this study. The animal study was reviewed and approved by the Committee for Experimental Animal Studies of Central South University.

## Author Contributions

TW performed the experiments. TW wrote the initial draft of the manuscript. TW, ZL, BT, TyW, JW, and FH contributed to the conceptual idea, the experimental design of the study, and the editing of the manuscript. All authors contributed to the article and approved the submitted version.

## Funding

This study was financially supported by the Provincial Natural Science Foundation of Hunan (2017JJ2336) and the Changzhutan National Independent Innovation Demonstration Zone special project (2017XK2108).

## Conflict of Interest

The authors declare that the research was conducted in the absence of any commercial or financial relationships that could be construed as a potential conflict of interest.
