# Supplementary material for: NEDD4 Induces K48-Linked Degradative Ubiquitination of Hepatitis B Virus X Protein and Inhibits HBV-Associated HCC Progression
Source: Front Oncol. 2021 Mar 9;11:625169. doi: 10.3389/fonc.2021.625169 (PMC7985090; doi:10.3389/fonc.2021.625169)
Supplement: Supplementary file 1 [file DataSheet_1.docx]

# Supplementary Table 1. Clinical pathological characteristics of 199 HCC patients.

| Characteristics | Number of class (%) | Characteristics | Number of class (%) |
| --- | --- | --- | --- |
| Age (year) |  | Gender |  |
| ≥60 | 96(48.2%) | Male | 155(77.9%) |
| <60 | 103(51.8%) | Female | 44(22.1%) |
| HBV infection |  | AFP (μg/L） |  |
| Positive | 104(52.3%) | ≥400 | 122(61.3%) |
| Negative | 95(47.7%) | <400 | 77(38.7%) |
| Child-Pugh |  | Histological grade |  |
| A | 179(89.9%) | G1 | 23(11.6%) |
| B | 17(8.5%) | G2 | 91(45.7%) |
| C | 3(1.5%) | G3 | 85(42.7%) |
| Tumor size |  | Microvascular invasion |  |
| ≥5 cm | 82(41.2%) | Yes | 116(58.3%) |
| <5 cm | 117(58.8%) | No | 83(41.7%) |
| TNM Stage |  |  |  |
| Ⅰ | 40(20.1%) |  |  |
| Ⅱ | 59(29.6%) |  |  |
| Ⅲ | 91(45.7%) |  |  |
| Ⅳ | 9(4.5%) |  |  |

# Supplementary Table 2. Sequences of siRNA and overexpression vectors used in this paper.

| **Gene** | **NCBI RefSeq** | **Sequence(5′-3′)** |
| --- | --- | --- |
| NEDD4 | NM_001284338 | https://www.ncbi.nlm.nih.gov/nuccore/NM_001284338.2 |
| HBX | NC_003977.2 | https://www.ncbi.nlm.nih.gov/nuccore/NC_003977.2?report=fasta&from=1376&to=1840 |
|  |  |  |
| **siRNA Sequence** | |  |
| NEDD4 siRNA | sense | AUUUGAACCGUAUAGUUCAGCdTdT |
|  | antisense | GCUGAACUAUACGGUUCAAATdTdT |
| Control siRNA | sense | UUCUCCGAACGUGUCACGUdTdT |
|  | antisense | ACGUGACACGUUCGGAGAAdTdT |

**Supplementary Figure 1**

**
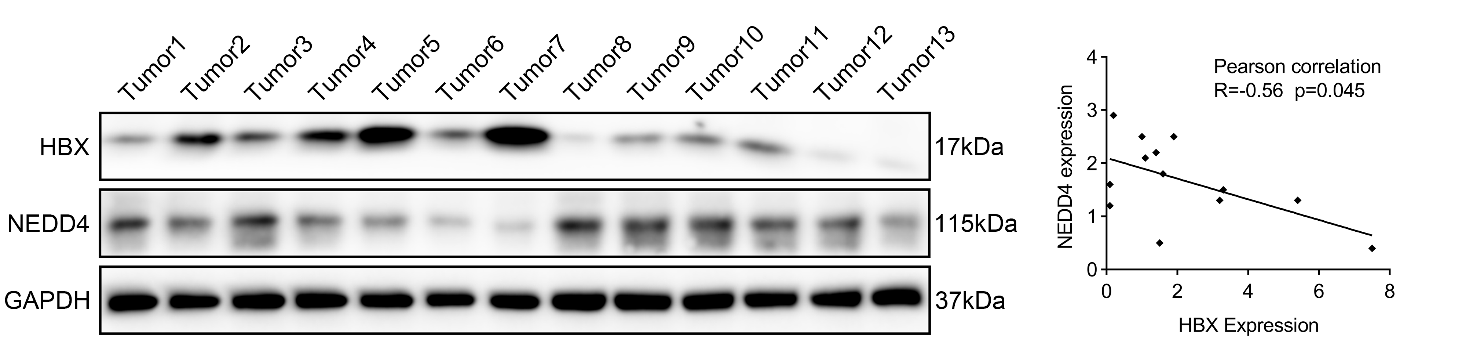
**

**Supplementary Figure 1. The correlation between HBx and NEDD4 expression in tumor tissues of HBV positive HCC patients.** We randomly selected thirteen tumor tissues of HCC patients with HBV infection to measure the protein levels of HBX and NEDD4. The results showed that the protein levels of HBX was inversely correlated with that of NEDD4.

**Supplementary Figure 2**

**
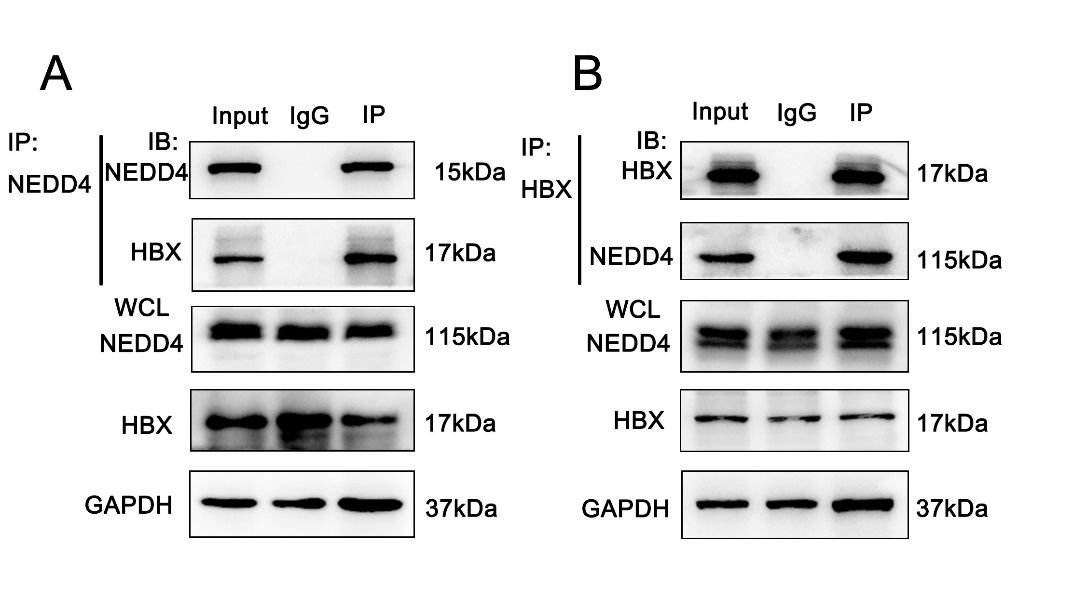
**

**Supplementary Figure 2. NEDD4 interacts with HBx in PLC/PRF/5 cells. (A)** and **(B)** Coimmunoprecipitation was conducted with NEDD4 and HBx antibodies. The coimmunoprecipitated mixture was separated by SDS-PAGE and evaluated by immunoblotting. NEDD4 and HBx interact with each other in PLC/PRF/5 cells.
